# Upper-Critical-Solution-Temperature Polymer Modified Gold Nanorods for Laser Controlled Drug Release and Enhanced Anti-Tumour Therapy

**DOI:** 10.3389/fphar.2021.738630

**Published:** 2021-09-23

**Authors:** Que Lin, Mao Jia, Yi Fu, Bei Li, Zhigang Dong, Xiaoya Niu, Zhen You

**Affiliations:** ^1^ Department of Head and Neck Oncology, West China Hospital of Stomatology, Sichuan University, Chengdu, China; ^2^ State Key Laboratory of Oral Diseases, West China College of Stomatology, Sichuan University, Chengdu, China; ^3^ Department of Orthodontics, West China Hospital of Stomatology, Sichuan University, Chengdu, China; ^4^ Department of Physiology and Pathophysiology, School of Basic Medical Sciences, Peking University, Beijing, China; ^5^ Department of Biliary Surgery, West China Hospital of Sichuan University, Chengdu, China

**Keywords:** gold nanorod, DOX, drug delivery, UCST polymer, photothermal therapy, tumor treatment

## Abstract

Photothermal therapy (PTT) has become effective method for the treatment of malignant cancer. The development of PTT system with high anti-tumour effect is still the feasible research direction. Here, a new type of gold nanorods (AuNRs)-doxorubicin (DOX)/mPEG_10K_-peptide/P(AAm-co-AN) (APP-DOX) nano drug delivery system was proposed. Among them, AuNRs was used as high-efficiency photothermal agent. APP-DOX had a suitable size and can be targeted to accumulate in tumour tissues through circulation in the body. The abundant matrix metalloproteinase 2 (MMP-2) in the tumour environment intercepted and cut off the short peptide chain structure grafted on APP-DOX. At the same time, the removal of the PEG segment leaded to an increase in the hydrophobic properties of the system. Nanoparticles aggregated into large particles, causing them to stay and aggregate further at the tumour site. When irradiated by 808 nm near-infrared laser, APP-DOX achieved a gradual heating process. High temperature can effectively ablate tumours and enable UCST polymer to achieve phase transition, resulting in more anti-cancer drugs loaded in the polymer layer DOX was released, effectively killing cancer cells. Animal experiments had verified the possibility of the nano drug-carrying system and good tumour treatment effect. What’s more worth mentioning is that compared with free DOX, the nano drug delivery system had lower biological toxicity and not cause obvious harmful effects on normal organs and tissues.

## Introduction

In the field of cancer treatment, traditional surgery, chemotherapy, and radiotherapy can cause long-term side effects and pain to patients (Choi et al., 2018). In contrast, photodynamic therapy (PDT), photothermal therapy (PTT), and chemodynamic therapy (CDT) have the advantages of noninvasiveness, higher selectivity, remote controllability, and lower biotoxicity (Li et al., 2020). Such methods have received increasing attention in the treatment of certain tumours. Among them, PTT, based on near-infrared (NIR) induction, converts light energy into thermal energy by virtue of photothermal conversion agents and thereby ablates tumours (Zhang et al., 2018; Hou et al., 2020). Commonly used photothermal conversion agents include small-molecule reagents (IR780, PcBu4 dye, and porphyrin) (Jung et al., 2018), gold nanomaterials [gold nanoparticles (AuNPs) and gold nanorods (AuNRs)] (Chuang et al., 2020; Rajkumar and Prabaharan, 2020), and graphene oxide (GO) (Deng et al., 2020). AuNRs have advantages over other gold nanomaterials due to their higher photothermal conversion efficiency and adjustable localized surface plasmon resonance (Yang et al., 2019; Wu and Dong, 2020). In addition, the biofriendly properties of AuNRs (including easiness of preparation and modification, lower toxicity, and higher tissue and organ tolerance) have promoted the application of AuNRs in the fields of biomedical diagnosis and treatment (Ge et al., 2019; Zheng et al., 2020). However, the small specific surface area of AuNRs has limited their drug-loading capacity, which is an urgent problem to solve (Zhang et al., 2012). So far, the functional modification of AuNRs and AuNR-based composite drug delivery systems (DDS) have been studied most. The materials commonly used to modify AuNRs include mesoporous silica (Ma et al., 2019), mesoporous manganese dioxide (Zhang and Ji, 2019), polydopamine (Cao et al., 2020), and hyaluronic acid (Gao et al., 2020).

In the past decades, DDS have been considered as an effective strategy to solve the problems of a short drug cycle and elimination by the mononuclear phagocyte system. DDS allows the release of active ingredients to target sites, decreases the required drug dose, reduces the side effects, and enhances the acceptance of new delivery systems by organs and tissues (Kingsley et al., 2006; Meng et al., 2019). Research on more intelligent and efficient drug delivery methods has been ongoing, among which the study of sensitive DDS stands out. Sensitive DDS can undergo physical or chemical phase transformations in response to environmental factor stimuli, such as temperature, pH, enzymes, and light (Ahmadi et al., 2020; Alvarez-Lorenzo et al., 2020; Ghaffar et al., 2020). For example, poly(N-isopropylacrylamide) (PNIPAM) is a typical sensitive polymer material because of its unique temperature sensitivity (its lower critical solution temperature (LCST) comes approximately 32°C) (Metawea et al., 2021). When the temperature is above the LCST, PNIPAM will undergo a sharp phase transition from a coiled hydrophilic conformation to a collapsed hydrophobic conformation (Liu et al., 2020; Yadav et al., 2021). Nevertheless, PNIPAM is not suitable for *in vivo* drug delivery due to its low biocompatibility and potential toxicity (Cooperstein and Canavan, 2013). Unlike LCST polymers, the upper-critical-solution-temperature (UCST) polymers used in biological applications also have good temperature-responsive ability. When the temperature is above their UCST, its polymer chains undergo phase transition from collapse to stretching (Asadujjaman et al., 2017). The UCST polymers provide a theoretical basis for drug loading and release. Specifically, P(AAm-co-AN), a copolymer of AN and AAm, and its derivatives are recent hotspots of UCST polymer research. By changing the feed ratio of AN and AAm, the UCST of the copolymer products can be finely adjusted to meet the requirements of various practical applications. For example, when the feed ratio of AN to AAm is in the range of 77.5:22.5–90:10, the UCST of the copolymer can be kept between 6.5°C and 66°C (Seuring and Agarwal, 2012; Asadujjaman et al., 2017; Otsuka et al., 2019).

Another often ignored issue of the DDS is the size of the drug-loaded nanomaterial, which affect tumour permeability, biodistribution, clearance from plasma and tissues, and metabolism *in vivo*. Nanomaterials with smaller sizes have better penetrating and inhibiting effects on tumour tissues (Ruan et al., 2015a). Although the small-molecule nanomaterials (<2 nm) can freely penetrate most tumour tissues, they fail to accumulate in the tumour tissues (Ruan et al., 2015b). Therefore, the drug-loading nanomaterials of variable size have also become a research hotspot.

**SCHEME 1:**
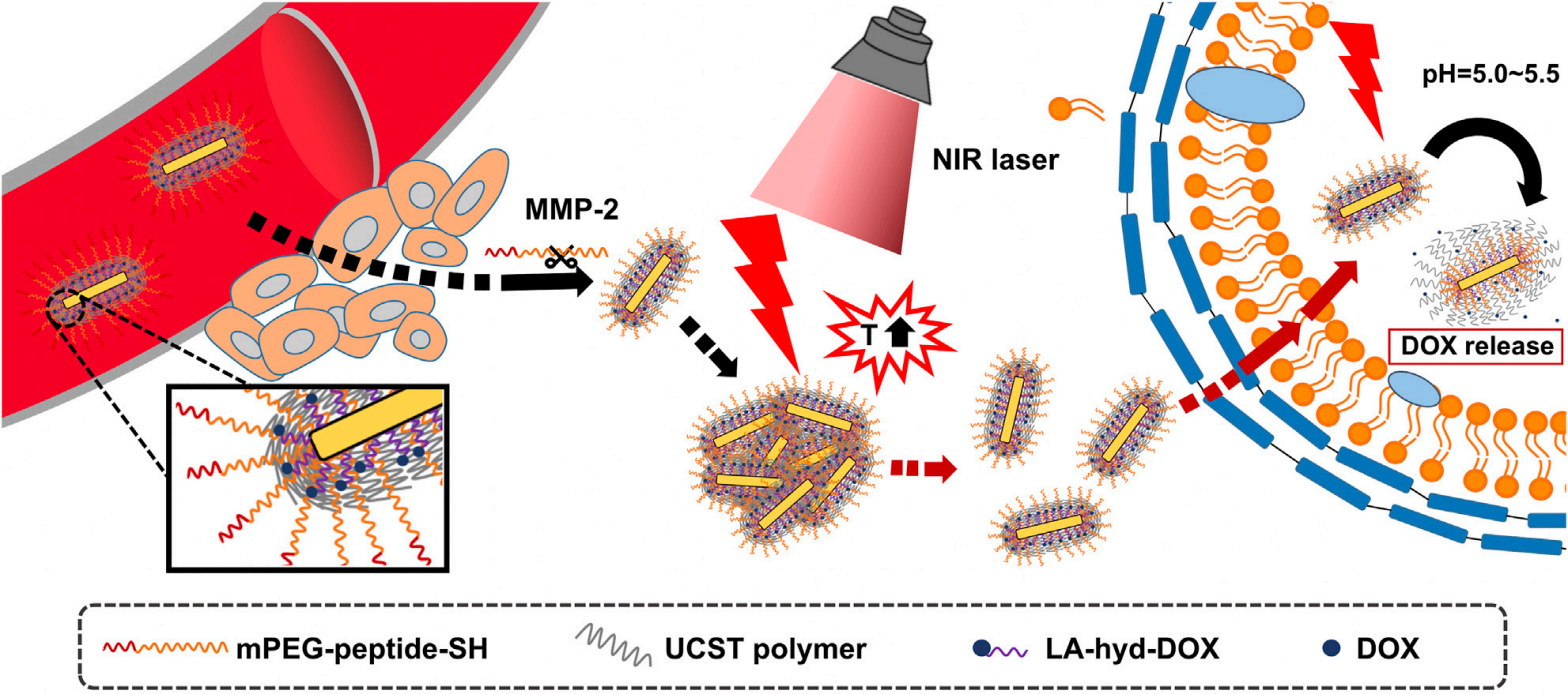
Illustration of the structure of APP-DOX and strategy of drug release at the tumour site.

In this study, we designed and constructed a size variable nanodrug delivery system with high photothermal conversion efficiency and good anti-tumour effect based on AuNRs and UCST polymers: AuNRs-DOX/mPEG_10K_-peptide/P(AAm-co-AN) (APP-DOX). As shown in Scheme 1, AuNRs were grafted with pH-sensitive group–modified doxorubicin (LA-hyd-DOX) and the UCST polymer (P(AAm-co-AN)-DDAT), as well as with matrix metalloproteinase 2 (MMP-2)-sensitive peptides, which then formed temperature-responsive vesicles through self-assembly. The UCST of P(AAm-co-AN)-DDAT was near 42°C, several degrees higher than human body temperature. After the systemic administration of APP-DOX, the delivery of APP-DOX to deep tumour areas was carried out in three steps. First, the APP-DOX reached the tumour tissue through the blood circulation. Second, APP-DOX penetrated the tumour vessel wall by the enhanced permeability and retention effect. APP-DOX exited the leaky vascular system and entered the tumour interstitium. Due to the high level of MMP-2 in the tumour, the MMP-2 sensitive peptide will be degraded to release the PEG segment, which results in the accumulation of APP-DOX inside the tumour. The thermal responsive ability of AuNRs was triggered by a NIR laser to achieve photothermal conversion and local heating. Meanwhile, the UCST polymer (P(AAm-co-AN)-DDAT), leading to the transformation of the larger APP-DOX aggregation into a smaller structure and causing the AuNRs to diffuse inside the tumour. In addition, the acidic microenvironment of the tumours triggered the continuous release of the model drug doxorubicin, thereby achieving the enhanced anti-tumour effect.

## Methods

### Materials and Animals

Tetrachloroauric acid (HAuCl_4_) and 2-(Dodecylthiocarbonothioylthio)-2-methylpropionic acid (DDAT) were purchased from Energy Chemical Company (Shanghai, China). Cetyl trimethyl ammonium bromide (CTAB), Acrylamide (AAm), and 2,2′-Azobisisobutyronitrile (AIBN) were obtained from Macklin Reagent Co., Ltd (Shanghai, China). Acrylonitrile (AN), Hydrochloric acid (HCl), L-Ascorbic acid (L-AA) and Methyl thiazolyl tetrazolium (MTT) were provided by Aladdin Reagent Co., Ltd (Shanghai, China). mPEG10k-GPLGIAGQC-SH was synthesized by Shanghai Apeptide Co., Ltd. (Shanghai, China). Doxorubicin hydrochloride (DOX·HCl) was provided by Dalian Meilun Biotechnology Co., Ltd (Liaoning, China). The HepG2 cancer cells were obtained from Nanjing KeyGen Biotech. Co. Ltd. (Nanjing, China). Female nude mice (18–22 g) were purchased from Qinglongshan animal breeding farm (Nanjing, China). All other reagents and solvents were purchased from Sinopharm Chemical Reagent Co., Ltd. (Shanghai, China).

### Synthesis of AuNRs

AuNRs with a length-to-diameter ratio of 4:1 was prepared using the classical seed-growth method (Zhang et al., 2017). Briefly, 7.5 ml of CTAB solution (0.1 M) was mixed with 250 µl HAuCl_4_ solution (10 mM) and the volume of the mixture was adjusted to 9.4 ml. Then, 0.6 ml NaBH4 solution (0.1 M) was added, and the mixture was shaken for 2 min to form the seed solution. In addition, 5 ml of HAuCl4 solution (0.001 M), 1 ml of AgNO3 solution (10 mM), 2 ml of HCl solution (0.5 M), and 800 µl of L-AA solution (0.1 M) were added successively to 100 ml CTAB solution (0.1 M), which was shaken for 5 min to form the growth solution. Afterwards, 500 µl of the seed solution was added to the growth solution described above. After 12 h of growth, the AuNRs were centrifuged twice at 9,000 rpm and then redispersed in deionized water for use.

### Synthesis of P(AAm-co-AN)-DDAT Polymer and Vesicles

P(AAm-co-AN)-DDAT was prepared by reversible addition-fragmentation chain transfer (RAFT) polymerization. In brief, 1.2 ml AN, 0.726 g AAm, 0.0098 g AIBN and 0.08 g DDAT (the molar ratio between AN, AAM and AIBN was about 310:170:1) were dissolved in 4 ml of 1,4-dioxane in a dry three-necked flask. The oxygen in the solution was removed by bubbling with nitrogen for 1 h. The reaction system was completely deoxygenated through three freeze–purge–thaw cycles. The three-necked flask was then transferred to an oil bath preheated to 65°C, where the reaction was carried out overnight for 18 h. The product was purified by reprecipitation three times in methanol. The final product, denoted P(AAm-co-AN)-DDAT, was obtained through lyophilization.

Self-assembled vesicles of P(AAm-co-AN)-DDAT were prepared using the membrane dialysis method. First, 10 mg P(AAm-co-AN)-DDAT was dissolved in 2 ml of mixed solvent, of tetrahydrofuran and dimethylformamide at a volume ratio of 2:3. Under magnetic stirring, 3 ml of deionized water was added dropwise to the mixture through an injection pump at a rate of 10 ml/h and mixed for 30 min. Lastly, the mixture was dialyzed in deionized water for 24 h to form vesicles of P(AAm-co-AN)-DDAT.

### Synthesis of LA-Hyd-DOX

LA-hyd-DOX was achieved through three steps. In detail, 0.35 ml of acetyl chloride was added dropwise to 50 ml of methanol and dispersed evenly by magnetically stirring for 5 min. Then 412.6 mg of α-lipoic acid was added to the above mixture. After uniform dispersion, the reaction was refluxed at 100°C for 8 h. The solvent was then removed by rotary evaporation. After freeze–drying, a yellow solid product, LA-oet, was obtained. After dissolving 300 mg of LA-oet in 30 ml ethanol, 10 ml of 85% hydrazine monohydrate was added to the solution and mixed thoroughly. The reaction was carried out under reflux at 80°C for 6 h. After the reaction was completed, the mixed solution was poured into ice water to produce LA-Hyd as a yellow solid precipitate. Next, 220 mg of LA-Hyd and 580 mg of doxorubicin hydrochloride were dissolved in 5 ml of anhydrous dimethyl sulfoxide. A drop of trifluoroacetic acid was added to the mixture, and the mixture was kept at 60°C for 24 h. Once the reaction was completed, the impurities were removed by reprecipitation three times in acetonitrile. The final product, LA-hyd-DOX, was obtained through lyophilization.

### Preparation of APP-DOX

First, 500 µl of 6 µM LA-hyd-DOX solution (pH 8.0) and 2 mg of mPEG_10K_-peptide-SH were added to 5 ml of 0.1 mg/ml AuNRs solution. The reaction mixture was stirred for 24 h in the dark. APP-DOX was prepared by membrane dialysis. Briefly, P(AAm-co-AN)-DDAT (10 mg) was dissolved in a mixed solvent (2 ml) of tetrahydrofuran and dimethylformamide at a volume ratio of 2:3. Under magnetic stirring, the mixture was added dropwise to the AuNRs solution through an injection pump at a rate of 10 ml/h. The mixture was dialyzed in deionized water for 24 h to remove the organic solvent, and the final product APP-DOX was obtained. AuNR-DOX and mPEG-AuNRs-DOX were prepared by AuNRs, La-hyd-DOX, P(AAm-co-AN)-DDAT and mPEG_10K_-SH in a similar way as above.

### Characterization

The morphology of the samples was examined in the JEM-1400 transmission electron microscope (JEOL Co., Ltd., Japan). Before TEM, the solution to be examined was diluted with deionized water and dropped onto a copper net. The particle size and zeta potential were monitored using the NanoBrook Omni laser particle size analyzer (Brookhaven Instruments, United States). Before the test, ultrasonic treatment was performed to ensure that the samples were evenly dispersed. The UV-vis absorption spectra were conducted on a 759S UV-vis spectrophotometer (Lengguang Technology, Shanghai).

### Drug Loading and Release

LA-Hyd-DOX or APP-DOX (10 mg) was transferred into a dialysis bags (MWCO = 3.5 kDa) and immersed in 50 ml of buffer solutions with pH values of 5.0, 6.8, and 7.4, respectively. At set time points, 3 ml of dialysate was regularly replaced with 3 ml of fresh buffer with the corresponding pH. The absorbance of the collected samples was measured in the UV-vis spectrophotometer. The cumulative release rates were calculated, and the release curves were plotted. Cumulative release was monitored by the same method in the different buffer solutions and under the different laser radiation conditions.

### Photothermal Performance

Using the LE-LS-808-XXTM infrared semiconductor laser device (Leoptics, Shenzhen), APP-DOX was irradiated with an 808 nm NIR laser under different power for various time. The temperature and the particle size changes of APP-DOX were recorded simultaneously using an electronic thermometer and NanoBrook Omni laser particle size analyzer (Brookhaven Instruments, United States), and the response curves were plotted. The thermal conversion efficiency of APP-DOX was calculated from the response curves.

### Cell Uptake

HepG2 cells were seeded into glass-bottom culture dishes at a density of 6×10^3^ cells per dish in 1 ml of DMEM medium containing 10% FBS, and cultured at 37°C with 5% CO_2_ for 24 h. After that, the culture medium was aspirated. The cells were overlaid with fresh culture medium containing various concentrations of mPEG-AuNRs-DOX, free DOX, and APP-DOX (100 µl per well) and cultured for 24 h. The cells were fixed with 4% paraformaldehyde for 25 min and washed three times with PBS. After staining the nuclei with Hoechst 33,342 for 25 min in the dark, the stained cells were washed thoroughly with PBS three times. Cell uptake was examined under the CKX53 inverted microscope (Olympus Corporation, Japan). The excitation and emission wavelengths were 480 and 590 nm, respectively.

### Cell Cytotoxicity

The toxicity of the nanocomposite material was tested *in vitro* using the MTT method. HepG2 cells were grown in a T-75 culture flask in DMEM medium with fetal bovine serum and 1% penicillin/streptomycin (containing 5% CO_2_) at 37°C for 24 h. Afterward, HepG2 cells were seeded into a 96-well culture plate at the density of 5×10^3^ cells per well for 24 h. Then, the culture medium was aspirated. The cells were overlaid with fresh culture medium containing different concentrations of pure AuNRs, mPEG-AuNRs-DOX, free DOX, and APP-DOX (100 µl per well) and cultured for another 24 h. Three replicate wells were set up for each concentration. After cocultivation, the corresponding control groups were irradiated with the 808 nm NIR laser under different power for various time. After irradiation, the culture medium was aspirated. The cells were washed three times with PBS, overlaid with 20 µl of 10 mg/ml MTT solution, and cultured for 4 h. The solutions in the wells were then completely aspirated. DMSO (150 µl) was added to each well to dissolve the crystals. The absorbance of each well was measured under the wavelength of 570 nm in the RT-6000 microplate analyzer (Rayto Life Science, Shenzhen), and the cell viability (%) was calculated from the absorbance.

### Animal Model and Drug Treatment

To establish a tumour model, 2×10^6^ HepG2 cells (in 200 µl of serum-free DMEM) were injected subcutaneously into the armpit of each female nude mouse. When tumour volume reached 200–300 mm^3^, the nude mice were randomly divided into seven groups of five and subjected to *in vivo* experiments. The mice were injected with 200 µl of mPEG-AuNRs-DOX, free DOX, or APP-DOX through the tail vein at a dose of 5 mg/kg of DOX. Mice injected with PBS served as the control group. After 8h, the nude mice were subjected to abdominal anesthesia using 0.1 ml of 5% chloral hydrate. The tumour areas were irradiated with the 808 nm NIR laser (1 w/cm^2^) for 10 min. The body weight and tumour volume of each group of nude mice were recorded at various times (0, 2, 4, 6, 8, 10, 12, 14, and 16 d). Tumour volume was measured with a Vernier caliper and calculated according to the formula. Tumour volume (mm^3^) = 0.5 × length × width (Li et al., 2020). In addition, the survival of the nude mice was recorded. The nude mice were sacrificed at 16 d. The tumour tissues were collected, weighed, and imaged.

### Histological Assays

The tumour tissues were collected from the nude mice that were sacrificed at 16 d, fixed with 4% paraformaldehyde for 2 d, embedded in paraffin, and sectioned with a microtome. Each sample was sliced into 5 µm sections and stained with hematoxylin–eosin (H&E). Finally, tissue damage was examined under an inverted fluorescence microscope. To examine the toxic effect of the nanomaterial on the organs of the nude mice, the main organs of the nude mice (heart, liver, spleen, lung, and kidney) were sectioned and H&E-stained as above. The sections were then observed and imaged under the inverted fluorescence microscope.

## Results and Discussion

### Synthesis and Preparation of APP-DOX

**FIGURE 1:**
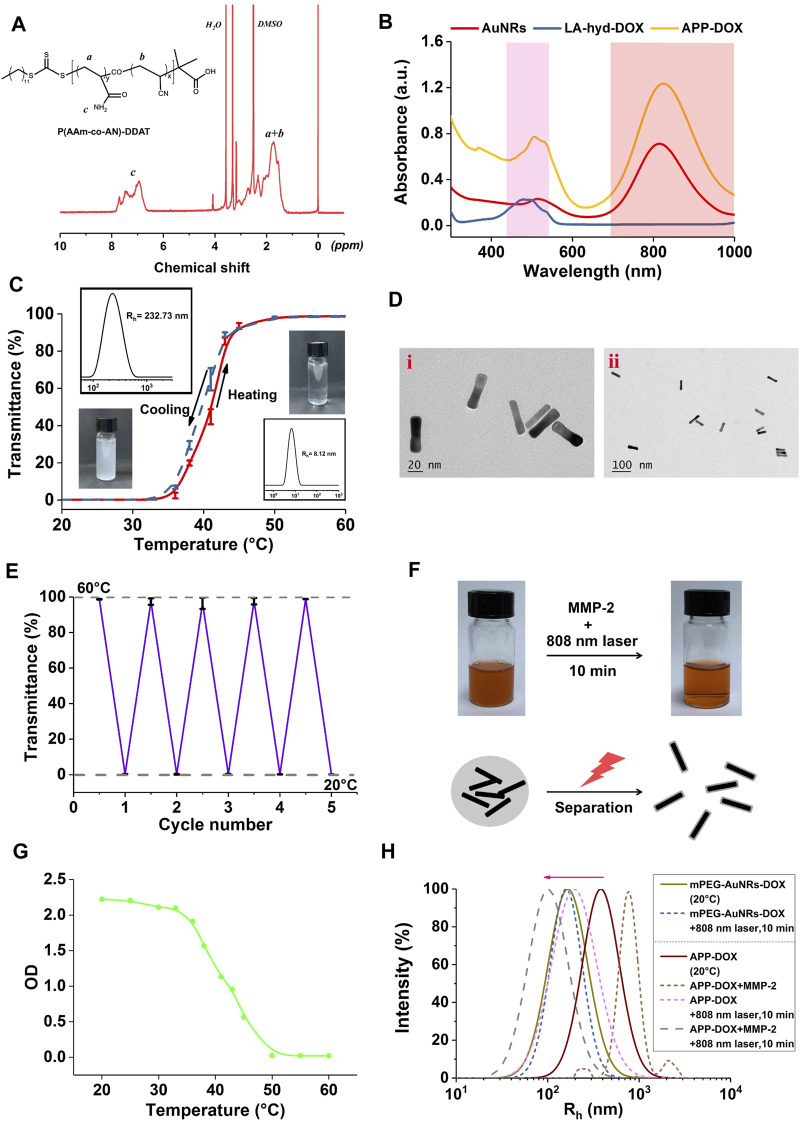
^1^H-NMR spectra of P(AAm-co-AN)-DDAT **(A)**. UV-Vis absorption spectra of AuNRs, LA-hyd-DOX, and APP-DOX **(B)**. Light transmittance-temperature curve, particle size distribution, and photographs of the self-assembled vesicles of the P(AAm-co-AN)-DDAT **(C)**. TEM images of AuNRs and APP-DOX **(D)**. Light transmittance curve of the self-assembled vesicles of the P(AAm-co-AN)-DDAT copolymer under alternate cycles of 20 and 60°C **(E)**. Photographs and micromechanism diagram of the AuNR-DOX turbidity change under 808 nm laser irradiation for 10 min **(F)**. Curve of the change in the absorbance (optical density value) of AuNR-DOX during the heating process from 20 to 60°C **(G)**. Particle size distribution curves of mPEG-AuNRs-DOX, APP-DOX, and APP-DOX co-incubated with MMP-2 before and after 808 nm laser irradiation **(H)**.

The UCST polymer P(AAm-co-AN)-DDAT was synthesized via RAFT polymerization using DDAT as a chain transfer agent. The structure of the product was determined by nuclear magnetic resonance (NMR) spectrum. The ^1^H-NMR spectrum of P(AAm-co-AN)-DDAT is shown in Figure 1A. The UCST temperature of P(AAm-co-AN)-DDAT could be tuned by adjusting the molar ratio of AAm to AN in the product. The peaks in the range of 6.67–7.82 ppm belonged to the protons of the amino groups in the AAm component, while the peaks in the range of 1.19–2.40 ppm belonged to the protons of -CH_2_-CH- in the main chain of the polymer (Huang et al., 2019; Tian et al., 2019). According to the peak area ratio of the characteristic peaks in ^1^H NMR, the proportions of AAm and AN in the product were calculated to be 68.2 and 31.8%, respectively. That is, the molar ratio of AAm to AN was approximately 17:8 in the copolymer.

To explore the thermal responsiveness behavior of the P(AAm-co-AN)-DDAT polymer, UV-Vis spectrophotometry and dynamic light scattering technology were used to monitor the changes in the turbidity and hydrodynamic diameter of the self-assembled vesicle upon temperature changes. As shown in Figure 1C, when the temperature was 20°C, the P(AAm-co-AN)-DDAT chain self-assembled into vesicles with a diameter of 232.73 nm in the aqueous solution. At this temperature, the polymer solution was in a turbid state, and the light transmittance stayed near 0%. When the temperature rose to 60°C, the hydrodynamic diameter of the P(AAm-co-AN)-DDAT decreased to approximately 8 nm. At this temperature, the polymer solution was clear and transparent, and the transmittance was close to 100%. These results demonstrated that the prepared block copolymer could self-assemble into a vesicle structure in water and had a significant temperature-responsive behavior. Figure 1E displays the change in light transmittance within four cycles of temperature switching between 20°C and 60°C. The vesicles showed no relaxation after four cycles and still had good temperature response performance.

LA-Hyd-DOX was designed for rapid response to acidic pH values, and was synthesized by linking doxorubicin and the hydrazide derivatives of lipoic acid (LA). Supplementary Figures S1A–E shows the ^1^H-NMR spectra of LA, LA-oet, LA-Hyd, DOX, and LA-Hyd-DOX. The spectra results are consistent with previous studies (Wang et al., 2018a; Wang et al., 2018b), indicating the successful synthesis of LA-Hyd-DOX.

To prepare the APP-DOX drug-loading system, LA-hyd-DOX and mPEG_10K_-peptide-SH were first grafted onto AuNRs through the coordination bond between the sulfhydryl group and gold. The block copolymer P(AAm-co-AN)-DDAT was also connected to AuNRs *via* the sulfhydryl-gold coordination bond using the membrane dialysis method, and the drug-loading system self-assembled into a vesicle structure to protect the loaded DOX. APP-DOX without the modification of mPEG_10K_-peptide-SH was synthesized as a control (AuNR-DOX). Figure 1B shows the UV-Vis absorption spectra of AuNRs, LA-hyd-DOX, and APP-DOX. AuNRs displayed two characteristic peaks at 550 and 808 nm, corresponding to its transverse surface plasmon resonance and longitudinal surface plasmon resonance absorption peaks respectively. In addition, characteristic the absorption peak of DOX was observed in the spectrum of APP-DOX at the wavelength of 520 nm, which confirmed the successful conjugation of LA-hyd-DOX to AuNRs. The TEM image of AuNRs is shown in Figure 1D. The average length and width of AuNRs were 48.04 ± 0.96 nm and 12.08 ± 1.97 nm, respectively, that was, the aspect ratio was approximately 4:1. As shown in Figure 1F, AuNR-DOX without the modification of mPEG_10K_-peptide-SH solution experienced the transition from a turbid to a clear state when it was irradiated with the 808 nm NIR laser for 10 min. This indicates AuNR-DOX achieved the transition from agglomeration to dispersion in micromorphology. This transition was attributed to the photothermal transformation of AuNRs under laser irradiation, during which the NIR light was converted into heat energy and thus promoted the temperature rise of the solution system. When the temperature rose above the UCST of P(AAm-co-AN)-DDAT, the polymer chain segment underwent a phase transition from hydrophobic to hydrophilic status, resulting in the passive destruction of the vesicle structure (Asadujjaman et al., 2017). These hydrophilic P(AAm-co-AN)-DDAT chains on the surface of AuNRs would protect AuNRs from aggregation to dispersion state. Figure 1G shows the changes in the absorbance of AuNR-DOX during the process of heating from 20°C to 60°C, which further confirmed the variability in the turbidity of APP-DOX during the heating process. These results indicated that APP-DOX without the protection of PEG (AuNR-DOX) would form large aggregation. However, under the laser irradiation, AuNR-DOX would transform from large aggregates to dispersion state with small size (around 50–100 nm).

Figure 1H shows the hydrodynamic diameter distribution of mPEG-AuNRs-DOX and APP-DOX under different conditions. The particle size of mPEG-AuNRs-DOX changed slightly after being irradiated with the 808 nm NIR laser for 10 min, while APP-DOX showed an obvious trend of a size change due to the presence of P(AAm-co-AN)-DDAT. After APP-DOX was incubated with MMP-2 and irradiated with laser, the particle size of APP-DOX increased first (aggregation due to the release of PEG) and then decreased (because of the phase transmission of P(AAm-co-AN)-DDAT), which confirmed the mechanism of drug delivery proposed in this work. When APP-DOX entered the tumour stroma, MMP-2 degraded the short peptides in its structure, resulting in the departure of the PEG segment. The hydrophobicity of APP-DOX increased, which further accumulated at the tumour site. Afterwards, NIR laser irradiation triggered the thermal response capability of AuNRs, thereby realizing photothermal conversion and causing local heating. Inducing the extension of the P(AAm-co-AN)-DDAT polymer chain, AuNRs diffused within the tumour, resulting in the transformation of large APP-DOX into small structures.

### Photothermal Properties of APP-DOX

**FIGURE 2:**
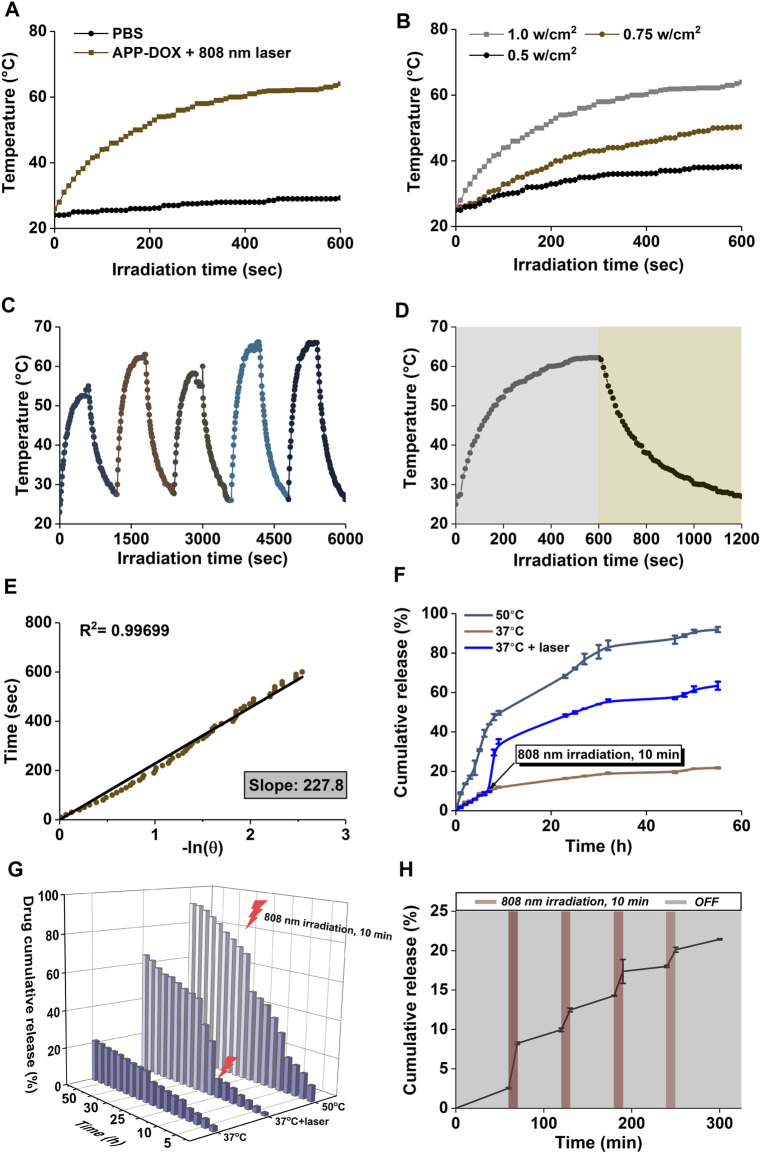
Temperature change curves of APP-DOX solution and the same amount of PBS under laser irradiation (808 nm, 10 min) **(A)**. Temperature change curves of APP-DOX solution under laser irradiation of different power densities **(B)**. Temperature change curves of APP-DOX solution during the on/off switching cycles of laser irradiation **(C)**. Temperature change curves of APP-DOX solution in a single on/off process of laser irradiation **(D)**. Relationship between the cooling time and the negative natural logarithm of temperature driving force after the laser was turned off **(E)**. Cumulative drug release curves **(F)** and the corresponding three-dimensional numerical histogram **(G)** of APP-DOX in pH 5.0 buffer at 37°C, at 50°C, and under the stimulation of NIR laser irradiation. Drug release curve of APP-DOX over 300 min of on/off cycles of NIR laser irradiation **(H)**.

As shown in Figure 1B, the absorption spectrum of the APP-DOX disclosed a broad-spectrum absorption in the UV-Vis light and even the NIR region, indicating that the system could be used as a good photothermal conversion agent for PTT. The photothermal conversion performance of the prepared APP-DOX was examined based on the solution temperature change under NIR laser irradiation. Figure 2A shows the temperature change curves of the APP-DOX solution and the same amount of PBS upon exposure to the NIR laser with a power of 1 w/cm^2^ at 808 nm. During the test, the real-time temperature was recorded every 10 s using an electronic thermometer. As shown in Figure 2B, APP-DOX exhibited good thermal response under laser irradiation. The temperature of the solution rose from room temperature to approximately 60°C within 10 min. Figure 2B illustrated that the solution could be heated to different temperatures by adjusting the power of laser. Figure 2C shows that APP-DOX maintained a good thermoresponsive capability in five heating–cooling cycles, and the temperature stayed above 50°C.

To calculate the photothermal conversion efficiency (η) of the drug-loading system, APP-DOX was irradiated with the 808 nm laser (1 w/cm^2^). After the temperature reached a plateau, the laser was removed and the solution was naturally cooled to room temperature. The temperature change curves are shown in Figure 2D. Figure 2E displays the cooling time plotted against the negative natural logarithm of the temperature driving force in this process. The data shows a positive correlation, with a coefficient of determination R (Li et al., 2020) = 0.99699, indicating a high degree of linear fitting. The photothermal conversion efficiency (η) of APP-DOX was calculated according to the following Eq. 1 (Wang et al., 2020).

$$\eta =\frac{\left[\text{hs}\left({\text{T}}_{\text{max,NP}}{-\text{T}}_{\text{surr}}\right){-\text{Q}}_{\text{dis}}\right]}{\text{I}\left({1-10}^{-\text{A}808}\right)}$$ (1)

where h was the heat transfer coefficient, s was the surface area of the container, T_max_ was the maximum temperature of the solution, T_surr_ was the ambient temperature, I was the laser power density, A808 was the absorption value of the material at 808 nm, and Q_dis_ was the heat generated by water and the container after absorbing light. To calculate hs, the following Eqs 2, 3 was used.

$${\text{Q}}_{\text{dis}}=\text{hs}\left({\text{T}}_{\text{max,H}2\text{O}}{-\text{T}}_{\text{surr}}\right)$$ (2)

$$\tau s=\frac{{\text{m}}_{\text{D}}{-\text{c}}_{\text{D}}}{\text{hs}}$$ (3)

where m_D_ was the quality of water; c_D_ was the heat capacity of water (4.2 J/g/°C); and τs was the time constant of the sample system, which was calculated according to the following Eqs 4, 5.

t=-τslnΘ (4)

$$\Theta =\frac{\left({\text{T}}_{\text{surr}}-\text{T}\right)}{\left({\text{T}}_{\text{surr}}{-\text{T}}_{\text{max}}\right)}$$ (5)

The photothermal conversion efficiency of APP-DOX (η) was calculated to be 33.9% according to the above formula. The above results indicate that APP-DOX has broad application prospects in PTT in the NIR biological window due to its stability and good photothermal capability.

### Drug Loading and Release

Supplementary Figures S1F–H displays the cumulative release process of DOX in different pH buffers. The 55-h cumulative release ratio of LA-Hyd-DOX were 22.17, 32.11, and 80.71%, respectively, in pH 7.4, 6.8, and 5.0 buffers. These results indicate that LA-hyd-DOX had a higher cumulative release efficiency under acidic pH and the behavior of LA-hyd-DOX was controlled by the acid-cleavable hydrazone bond (-NH-N = ). Acidic pH–sensitive LA-hyd-DOX would be beneficial to targeted cancer therapy, as it could significantly reduce the premature release of drug in blood circulation (pH = 7.4). When the nanomaterials were internalized and entered the endocytic chamber (pH < 6.5), they continued to release drug to kill tumour cells.

Figures 2F,G show the cumulative drug release curves and the corresponding three-dimensional histogram of APP-DOX in pH 5.0 buffer at 37°C and 50°C as well as under the stimulation of NIR laser irradiation, which simulated the *in vivo* drug release process of the drug-loading system at the tumour location. The 55-h cumulative release ratio of APP-DOX in 37°C and 50°C buffers were 21.76 and 91.92%, respectively. The significant difference in release ratio was attributed to the high temperature–induced phase transition of the P(AAm-co-AN)-DDAT polymer segment in the APP-DOX structure, which promoted the exposure of the LA-hyd-DOX segment to the acidic environment. The acidic environment continued to induce the breaking of the acid-sensitive hydrazone bond (-NH-N = ) in the LA-hyd-Dox structure, thereby releasing the DOX continuously. Figure 2H shows the drug release curve of APP-DOX under the on/off switching of NIR laser irradiation within 300 min. The results further demonstrated that APP-DOX had stable and good photothermal performance and that its drug release process could be regulated by controlling the laser irradiation.

### Cellular Uptake

**FIGURE 3:**
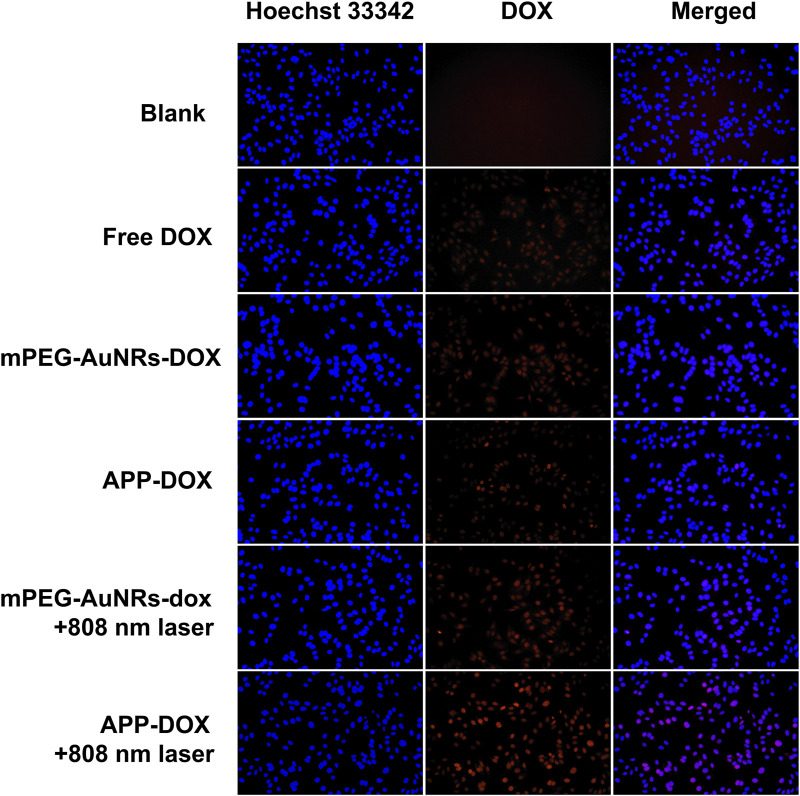
Fluorescence microscopic images of HepG2 cells incubated with free DOX, mPEG-AuNRs-DOX, and APP-DOX for 4 h (scale bar represents 10 µm).

Figure 3 shows the fluorescence microscopic images of the endocytosis of free DOX, mPEG-AuNRs-DOX, and APP-DOX by HepG2 cells. In the absence of laser irradiation, HepG2 cells showed weak red fluorescence (DOX), indicating that only a small amount of DOX internalized by cells. When APP-DOX was irradiated with the 808 nm NIR laser, the red fluorescence was significantly enhanced, indicating that the photothermal effect of AuNRs significantly enhanced the drug release of APP-DOX and promoted the uptake by HepG2 cells.

### Cytotoxicity

**FIGURE 4:**
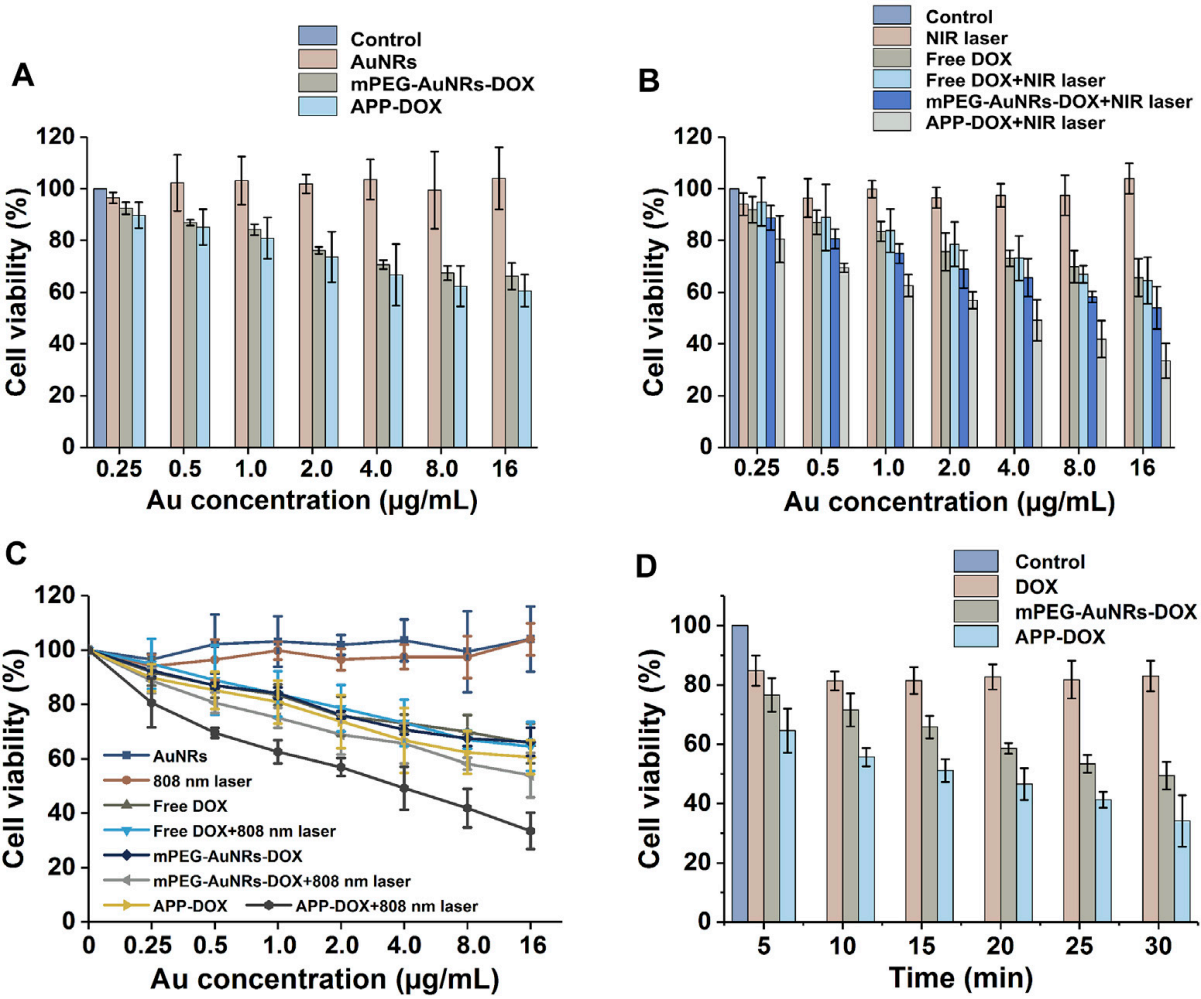
Numerical histogram **(A,B)** and broken-line graph **(C)** of HepG2 cells viability after treatment with nanomaterials under laser irradiation and the histogram of the relationship between laser irradiation time and cell survival rate **(D)**.

The PTT efficacy of APP-DOX was evaluated by the MTT *in vitro* cytotoxicity assay. Figure 4 shows the histogram of cell viability of HepG2 cells after coincubation with nanomaterials under different conditions. Pure AuNRs, the main components of the nano-drug-loading system, had no significant cytotoxicity because they are inert inorganic materials with good biocompatibility. Although free DOX, mPEG-AuNRs-DOX, and APP-DOX all showed concentration-dependent cytotoxicity, their negative effect on cell viability was negligible at low concentrations. Comparing Figures 4A,B, APP-DOX showed significant toxicity toward HepG2 cells at high concentrations. Moreover, APP-DOX showed the highest cytotoxicity after being irradiated with the 808 nm NIR laser. HepG2 cells had a cell viability of 33.45% when the Au concentration of APP-DOX was 16 µl/ml upon 5 min laser irradiation. In contrast, the cell viability of other groups of cells at this Au concentration (16 µl/ml) all exceeded 50%. A broken-line trend chart (Figure 4C) was drawn based on the corresponding values in Figures 4A,B, and it shows more intuitively the high cytotoxicity of APP-DOX.

Figure 4D explores the relationships between the laser irradiation time and the cell viability corresponding to DOX, mPEG-AuNRs-DOX and APP-DOX under irradiation. The effect of free DOX on cell viability was basically independent of laser irradiation. As the duration of laser irradiation increased, both mPEG-AuNRs-DOX and APP-DOX showed high cytotoxicity, which was attributed to the photothermal effect of AuNRs. Longer laser irradiation led to a greater heat accumulation, and the heating process resulted in excessive apoptosis of tumour cells. The APP-DOX group showed a lower cell survival rate than that of mPEG-AuNRs-DOX group under same condition. For example, the viability of APP-DOX group was 34.13% after laser irradiation for 30 min, whereas the mPEG-AuNRs-DOX group had a viability of 49.43%. Apparently, the lower viability could be attributed to the combination of the photothermal effect of AuNRs and enhanced cellular uptake.

### Antitumour Effect

To evaluate the efficacy of PTT against solid tumours, a tumour model was established through subcutaneous inoculation of nude mice with HepG2 cells. When the tumour volume increased to approximately 200–300 mm^3^ in nude mice, the nude mice were randomly divided into seven groups of five. The mice were injected with 200 µl of mPEG-AuNRs-DOX, free DOX, or APP-DOX through the tail vein at a dose of 5 mg/kg of DOX. At 8 h after injection, the tumour areas were irradiated with the NIR laser. The day of injection was set as 0 d, and the tumours were then observed for 16 days.

**FIGURE 5:**
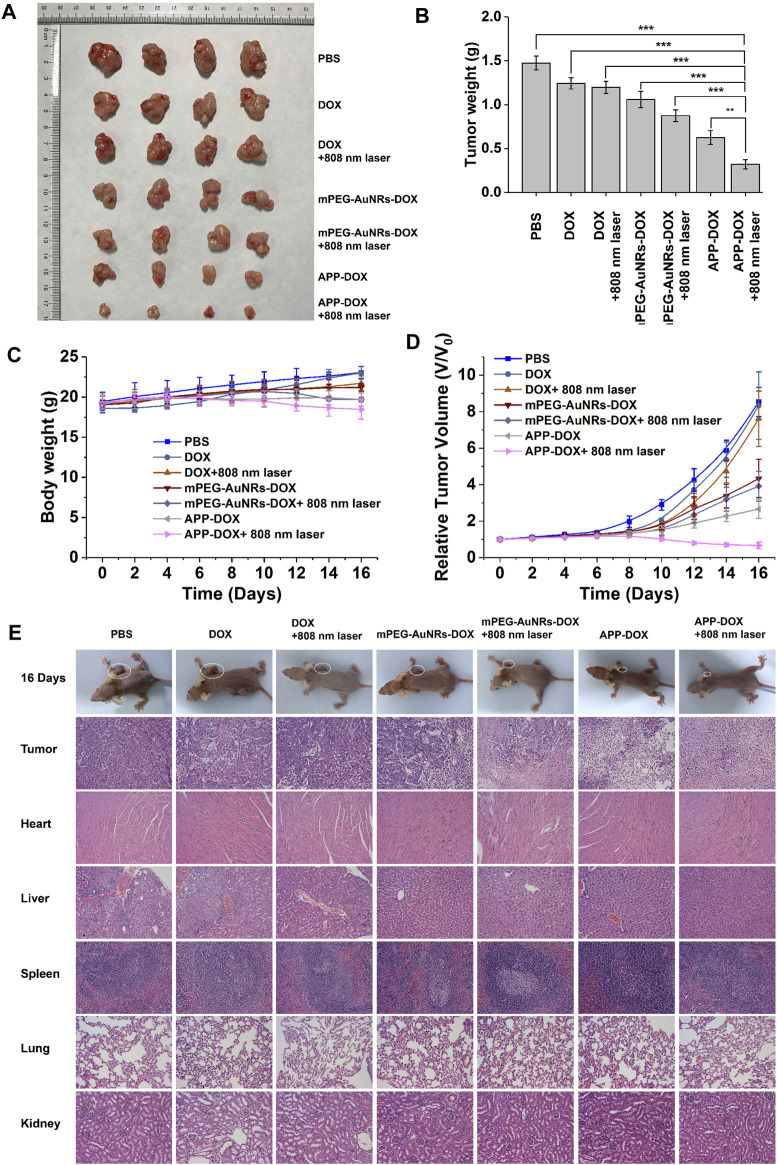
The efficacy of PBS, DOX, mPEG-AuNRs-DOX, and APP-DOX in treatment of subcutaneous hepatocellular carcinoma xenograft tumours in nude mice. Images of the tumours in nude mice **(A)**. Bar graph of tumour weight in each group of nude mice **(B)**. Weight change curves of the various groups of nude mice **(C)**. Curves of the relative tumour volume change **(D)**. Photographs of nude mice and H&E staining images of the pathological sections of tumours and main organs (heart, liver, spleen, lung, and kidney) **(E)**.

Figure 5C shows the body weight of all groups of nude mice at different time. None of the groups showed a significant decrease in body weight, indicating that the free drugs and nanomaterials had no acute toxicity with the administered dose. The relative changes in tumour volume are plotted in Figure 5D. Compared with the PBS group, the DOX group and the DOX+808 nm laser group only showed weak anti-tumour effect in the first 10 days, while no obvious inhibitory effect on tumour growth was observed thereafter. The average tumour volume of PBS, DOX and DOX+808 nm laser had increased approximately 8 times than the original size on 16 d. In contrast, the APP-DOX group and the APP-DOX+808 nm laser group showed a significant therapeutic effect and caused the ablation of the solid tumours. In the APP-DOX+808 nm laser group, the tumour volume was decreased to 0.6 times of the original size on 16 d. These results indicate that APP-DOX displayed a better drug-releasing and anti-tumour effect under the photothermal effect of AuNRs. Although the permanent and complete elimination of tumour tissue had not been achieved in this group, the therapeutic effect was significant, and the residual tumour tissues could be removed by surgery. After treatment for 16 d, the nude mice were sacrificed. The tumour tissues were removed, weighed, and imaged. The photographs and the histogram of tumour weight are shown in Figures 5A,B. Tumour weight and size were significantly smaller in the APP-DOX+808 nm laser group than other groups.

The tumour tissues and other organs were sliced into 5 µm sections, which were subjected to H&E staining. The results of histopathological examination are shown in Figure 5E. The group injected with PBS showed the typical pathological characteristics of tumours. Namely, there were many proliferative cancer cells in the tissues, which had large and irregular nuclei and a small amount of cytoplasm (Thoenes et al., 1986). The DOX group and the DOX+808 nm laser group resembled the PBS control group. The mPEG-AuNRs-DOX group had fewer cancer cells, and the cancer cells showed nuclear pyknosis. In the mPEG-AuNRs-DOX+808 nm laser and the APP-DOX groups, the number of cancer cells in the fields of view was further reduced. Nuclear pyknosis was further aggravated, and a gap appeared. Under the simultaneous action of heat and drug release, the APP-DOX+808 nm laser group showed the greatest decrease in the number of cancer cells and the largest gap formation after nuclear pyknosis compared with the other groups. These results indicate that APP-DOX had the strongest anti-tumour effect under the synergistic action of PTT and chemotherapy.

Sections of the main organs of nude mice (heart, liver, spleen, lung, and kidney) were subjected to H&E staining and histological examination. Except for the slight inflammation observed in the liver tissue of the PBS injection group, no significant damage was found in the main organs of the nude mice. These results demonstrated that the drug-loading nanoparticles prepared in the present study had low cytotoxicity. APP-DOX is the ideal candidate material for synergistic treatment of liver tumours with PTT and chemotherapy, and it has minimal toxic and side effects on normal organs.

## Conclusion

In summary, we have developed a unique drug delivery system APP-DOX based on AuNRs and UCST polymer for the treatment of tumours. MPP-2 can intercept and cleave short peptides in the APP-DOX structure, and increase the hydrophobicity of the nanoparticles, leading to the further accumulation of APP-DOX at tumour site. In addition, APP-DOX exhibited excellent light-to-heat conversion efficiency, and can achieve a gradual increase in temperature under NIR laser irradiation. Local high temperature can kill cancer cells and ablate tumour tissue. When the temperature was higher than the UCST of P(AAm-co-AN), it triggered its phase transition, resulting in the faster release of the loaded anti-tumour drug DOX, and achieving high-efficiency anti-tumour performance. The *in vivo* anti-tumour results of APP-DOX showed that it could effectively inhibit tumour growth without obvious physiological toxicity.

## Data Availability

The raw data supporting the conclusion of this article will be made available by the authors, without undue reservation.
