# Supplementary material for: Upper-Critical-Solution-Temperature Polymer Modified Gold Nanorods for Laser Controlled Drug Release and Enhanced Anti-Tumour Therapy
Source: Front Pharmacol. 2021 Sep 23;12:738630. doi: 10.3389/fphar.2021.738630 (PMC8495017; doi:10.3389/fphar.2021.738630)
Supplement: Supplementary file 1 [file Image1.pdf]

Supporting Information

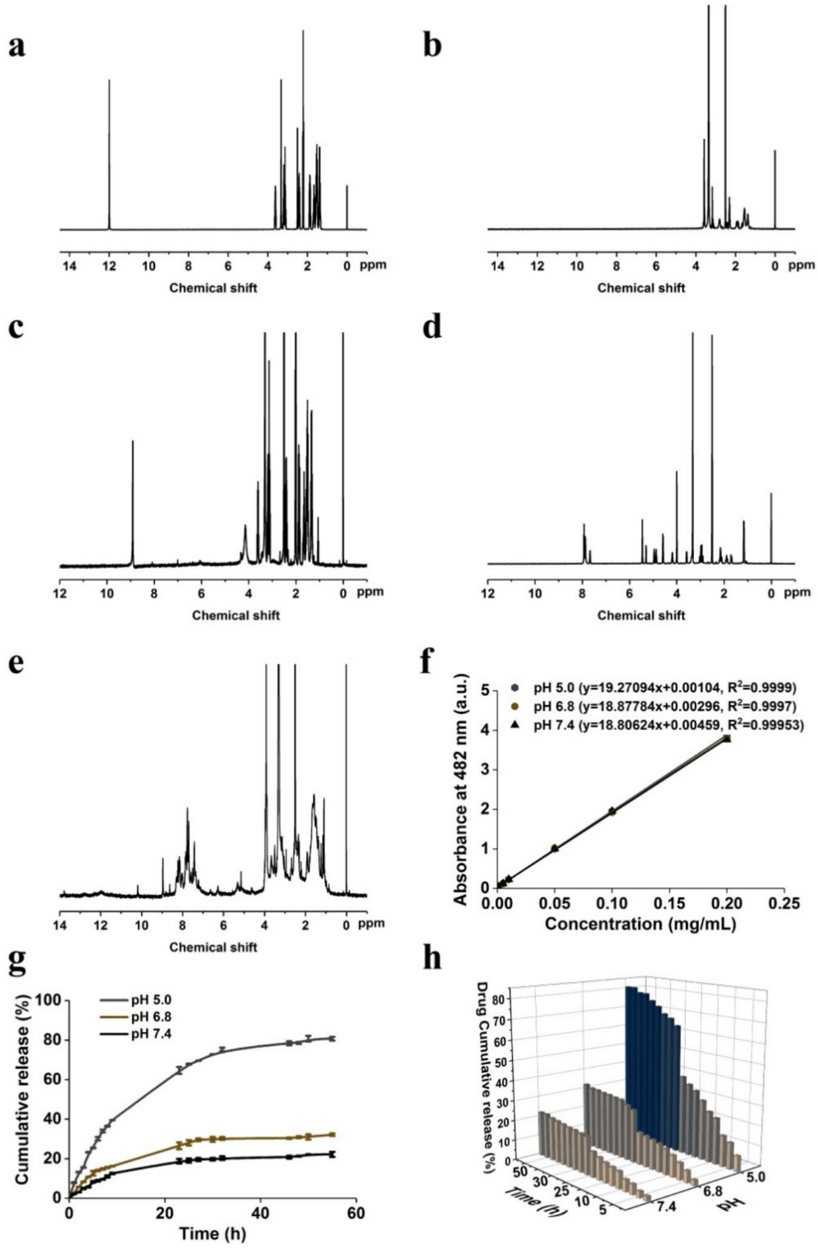

Figure s1 ^{1}H-NMR spectra of LA (a), LA-oet (b), LA-Hyd (c), DOX (d), and LA-Hyd-DOX (e). Standard curves of LA-Hyd-DOX in pH 5.0, 6.8, and 7.4 buffers (f) (concentration range 0-0.2 mg/mL). Curves (g) and numerical histogram (f) of the cumulative release of LA-Hyd-DOX in pH 5.0, 6.8, and 7.4 buffers.
